# Video Game Addictive Symptom Level, Use Intensity, and Hedonic Experience: Cross-sectional Questionnaire Study

**DOI:** 10.2196/33661

**Published:** 2022-06-09

**Authors:** Bhavneet Walia, Jeeyoon Kim, Ignatius Ijere, Shane Sanders

**Affiliations:** 1 Department of Public Health Falk College Syracuse University Syracuse, NY United States; 2 Department of Sport Management Falk College Syracuse University Syracuse, NY United States

**Keywords:** video game use, addictive behaviors, mental health, video game addiction, videogames, addiction, video games

## Abstract

**Background:**

The effects of behavioral addiction to video games has received increasing attention in the literature, given increased use intensity among subgroups of video game players.

**Objective:**

This study seeks to empirically determine the relationship between intensity of video gaming and hedonic experience of the player.

**Methods:**

We conducted a survey of 835 individuals who regularly play video games to determine the relationship between intensity of use and hedonic experience. We divided the sample into quartiles by self-reported video game addictive symptom level (from the Internet Gaming Disorder Scale) and conducted polynomial regressions separately for each quartile.

**Results:**

We found that the higher video game addictive symptom level groups experienced a U-shaped (curvilinear) relationship between hedonic experience and intensity of play, whereas groups with lower video game addictive symptom levels exhibited no such relationship. The coefficients for the highest addictive symptom level group (quartile 4) for marginal effects for hours played per week and hours played per week squared were significantly negative (*P*=.005) and significantly positive (*P*=.004), respectively.

**Conclusions:**

Our results are consistent with sensitization and tolerance theories, which suggest that high-symptom groups experience frustration and disappointment until they achieve excessive dopamine release, at which point their hedonic experience is expected to improve with additional play. Conversely, low-symptom groups experience no such fall-and-rise pattern. This result is consistent with the outcome that members of the latter group play the game for the direct experience, such that their hedonic experience is more directly related to events occurring in the game than to the increasingly elusive pursuit of excessive dopamine release. We also find that high-symptom groups spend substantially more time and money to support video game use and are much more likely to engage in video game use at the expense of other important activities, such as working, sleeping, and eating.

## Introduction

In our current technological era, video games have become pervasive in the United States and many other developed countries. This form of gaming has high prevalence among people of different ages, socioeconomic levels, and cultural environments. The Entertainment Software Association (ESA) reports that “we are living in the golden age of video games, and video game players are thriving” [1]. In 2020, the ESA found that 75% of Americans had a gamer in their household and 64% of American adults played video games. A primary appeal of video games is their ability to provide pleasure or mental stimulation to players [2]. These attributes, along with technological improvements allowing for remote competitive play, have helped to drive the observed high rate of growth in video game participation. In general, video game players can vary widely in terms of intensity and competitive level [3,4]. In 2020, the ESA reported that 80% of video game players said the activity provided mental stimulation, and 79% said they experienced high levels of relaxation [1].

Video games are not only pervasive in many areas but also potent enough to provide hedonic experiences to some players. The pursuit of this hedonic experience has caused many players to make video games an essential routine in their lives. Video games have become a booming component of the entertainment industry; however, some health issues have been associated with them [5]. Studies have identified negative consequences of chronic play, which include social, professional, and educational impairment [6]. Hence, very much like substance use disorders, video game disorder is a “persistent or recurrent behavior pattern of sufficient severity to result in significant impairment in personal, family, social, educational, occupational or other important areas of functioning” [3]. Consequently, in 2018, the World Health Organization included video game disorder as an essential type of mental health disorder [3]. This is in accord with the fifth revision of the Diagnostic and Statistical Manual of Mental Disorders.

Many studies have been conducted on the psychological components of video games; others have concentrated on the “emotional states of the game player” [6] and the “playful-consumption experience of videogame[s]” [7]. From the hedonic perspective, the Greek philosopher Aristippus wrote that a good life is “to experience the maximum amount of pleasure, and that happiness is the totality of one’s hedonic moments.” From this perspective, the pursuit of hedonic enjoyment though the attainment of pleasure and avoidance of pain improves one’s well-being as subjectively assessed based on cognitive and affective evaluation of one’s perceived happiness in life [8]. In this sense, the term “subjective well-being” (or alternatively, “hedonic well-being”) is widely used in attempts to understand how hedonic enjoyment contributes to well-being. Hedonic enjoyment increases positive affect, decreases negative affect, and heightens life satisfaction [9]. Although construing well-being based on pleasure or happiness provides a useful understanding of self-perceived psychological states, the subjective well-being approach has limitations in that pleasurable experiences are not necessarily optimal in promoting wellness (eg, drug use or alcohol consumption). Experiencing pleasure (or hedonic value) is a key motive for video gaming [10]. When pleasure is the motivation, the pursuit of hedonic value becomes the end goal; the pursuit of pleasure can lead to enhanced happiness and hedonic well-being (ie, the aspect of well-being related to pleasure) but can also lead to perverse elements of learned addictive behavior over time [11]. Griffiths [12] suggests mood modification (ie, “the subjective experience that people report as a consequence of engaging in the particular activity”) and tolerance (ie, “the process whereby increasing amounts of the particular activity are required to achieve the former effects”) are common components of addiction; such components have been confirmed to be present in the video gaming context [13]. Tolerance indicates that the more one is exposed to a stimulus, the greater becomes the threshold for a rewarding experience [14].

The present study examines tolerance in the context of video games. Previous work has shown that excessive players of video games tend to be more reward dependent (for dopamine) [15]. Previous research has also found that when reward expectations for dopamine are not met, disappointment can be experienced (eg, in those with internet addiction disorder) [16,17]. Sensitization (ie, hypersensitive reaction to a stimulus) is another common response among addicted individuals, according to the literature [18,19]. That is, addicted individuals are more responsive to stimuli that trigger their need to engage in the behavior of interest. Hypersensitive response and stimulation make self-regulatory behaviors more challenging for these individuals, even when the consequences of failed self-regulation are not consistent with long-term well-being; time-consuming addictive behaviors can contribute to loss of priorities, sleep deprivation, or job loss. Further, research finds that individuals with behavioral addictions act as they do because the activity in question releases excessive levels of dopamine in the brain [20]. The behavior or activity increasingly becomes a vehicle for dopamine release rather than chiefly an activity in and of itself. The combination of tolerance and sensitization can create complementary difficulties for addicted individuals. Sensitization makes individuals more responsive to stimuli that trigger a behavior, whereas tolerance increases the intensity of the activity needed to achieve the desired release of dopamine [12,13]. The result is something of a trap: addicted individuals are easily triggered to begin the activity but must engage in the activity with increasing intensity to achieve the desired outcome. As the desired outcome relates less to the activity and more to its effects, moreover, individuals may not enjoy the activity until the desired cognitive effect is achieved.

Within the context of these prior research findings, we hypothesized that video game players who show addictive symptoms would experience a U-shaped relationship between hedonic experience in video games and video game hours played per week. If this relationship was present, then we would observe a significant negative association with video games and a significant positive association with video games squared, as in a standard polynomial regression analysis. Sensitization would increase the likelihood that such video game players would initiate play even when it does not improve quality of life (eg, even when the person has other pressing life priorities). As these types of video game players play additional hours on average, we expect that an individual’s hedonic response to the game will decrease with play intensity up to the point of excessive release of target levels of dopamine. Once video game play is initiated, we expect such users to be increasingly frustrated until they attain excessive dopamine release, due primarily to tolerance. Upon attainment of this target, however, we expect hedonic response will increase in play intensity. For video game users not experiencing addictive symptoms, we hypothesized that there would be no such U-shaped relationship. Rather, we expected such users to derive hedonic experiences directly from features of the game rather than from chasing a dopamine response. As such, we hypothesized that there would be a comparatively flat relationship between hedonic experience and video game hours played per week for this latter group of video game players.

To test this hypothesis, we divided the study subjects into quartiles based on self-reported addictive video game symptom levels using the Internet Gaming Disorder Scale (IGDS). Quartiles are commonly used in applied statistics to avoid arbitrary or convenient partition points. This method breaks the data into balanced subsamples by definition, because cutoffs are not arbitrarily determined by the researcher. Bennette and Vickers [21] state that “in contemporary epidemiologic practice, continuous variables are typically categorized into tertiles, quartiles and quintiles as a means to illustrate the relationship between a continuous exposure and outcome.” This approach was further validated by Maggiore et al [22], Kjos et al [23], and Sousa et al [24]. Good alternative methods of subsampling exist, such as the method employed by Zhu et al [25] to define categories for leisure gamers, excessive gamers, and pathological gamers based on cutoff values.

In our study, quartile 1 reported the lowest level of symptoms (mean symptom score 1.38) and quartile 4 reported the highest (mean symptom score 3.69). We used separate polynomial regression analyses for each quartile to test for significant nonlinear relationships between hedonic experience and video game hours played. Our use of polynomial regression to test for curvilinear relationships is a key contribution of this study, given that previous regression analyses of this topic have used linear regression. We studied this U-shaped relationship as a potential indicator of sensitization and tolerance in the addicted individuals. We specified a statistical model to explore this and, more specifically, estimated the “tipping point” at which the typical video game player exhibits addictive symptoms, such as attaining a target dopamine response and subsequently facing an increasing hedonic experience given additional video game play.

## Methods

### Overall Aims

This study aimed to examine how intensity of video game play (ie, casual or heavy usage) moderates the relationship between time spent on playing video games and hedonic experience. First, we hypothesized that subjects reporting higher behavioral addiction symptoms would show a U-shaped relationship between time spent on video games and hedonic experience. Second, we hypothesized that subjects reporting lower behavioral addiction symptoms would not show a U-shaped relationship between time spent on video game and hedonic experience, but instead would show a comparatively flat relationship.

### Recruitment

To test the hypotheses, an online survey-based study was conducted after acquiring approval from the institutional review board at Syracuse University (IRB number: 19-186). Based on several screening questions (eg, to determine use and knowledge of video games), subjects who played video games on a regular basis were identified and allowed to participate in the survey study. The target population were United States adults who played video games on a regular basis. Respondents were recruited and the survey was distributed via Amazon Mechanical Turk. The initial request asked them to participate in a survey about well-being. The survey was developed with Qualtrics software (SAP) and was a 6-screen survey. Interested subjects answered questions about their daily lives, including 2 screening questions: (1) Do you play video games on a regular basis? (responses were either yes or no) and (2) How often do you play video games? (multiple responses were available). Subjects who answered “no” to the first question and “not at all” to the second question were automatically excluded. Additionally, respondents who chose video game genres that they played in a multiple-choice question were also asked to list the game titles they had played in that genre. Respondents who listed titles that did not match the genre were excluded.

### Data Summary

Of 1072 attempts, 835 (77.8%) participants passed the screening and completed the survey. The average age of the participants was 32 years. More details on the respondents are presented in Table 1. To examine different types of players more specifically, we grouped respondents into quartiles based on game addiction symptoms.

In Lemmens et al’s study [26], video games players who were categorized as “addicted/disordered” constituted less than 5% of video game players. Quartile 4, representing the highest quartile, corresponded closely to the optimal cutoff point for disordered gamers defined by Qin et al [27]. Their optimal cutoff for defining problem gamers was a total 9-item IGDS score of 32, whereas the lower bound in our quartile 4 was 28. Therefore, all video game players that would have been classified as problem gamers in Qin’s definition were included in our quartile 4, along with some additional gamers with scores in the range of 28 to 31, which is near Qin’s cutoff. Given this close correspondence, as well as the empirical precedent of generating quartile categorical variables discussed previously, we maintained our quartile dummies in the regression analysis to follow. We adopted 3 items from the popular self-report scale described by Keyes [28], which measures hedonic experiences, to estimate respondents’ hedonic experience in video games (these items measure interest in life, satisfaction in life, and happiness experienced in video game play on a 6-point Likert scale ranging from “not at all” to “very extremely”); these items have been applied to previous video gaming studies [29]. The Cronbach *α* of our data was .9. Time spent on video games was measured with a single item: “On average, how many hours per week do you play video games?” (answers were open ended, ranging from 1 to 70 hours per week). The IGDS, defined by Lemmens et al [26], was also employed. This scale assesses 9 criteria for internet gaming disorder: preoccupation, tolerance, withdrawal, persistence, escape, problems, deception, displacement, and conflict. This represents one of the most commonly used gaming addiction symptom scales (responses were on a 5-point Likert scale, ranging from “never” to “very often”); the mean score was employed to group respondents based on behavioral addiction symptoms. The Cronbach *α* was .91. Additional items to determine the level of neglect of other activities due to video games were also constructed. The first item was “Have you neglected school or work so that you could play games?” Additional items were phrased similarly, asking about neglect of sleeping, eating, socializing with others, and physical activity (responses were on a 5-point Likert scale, ranging from “never” to “very often”). Other items were used to examine money spent on video games (1 item, “How much money do you spend on video games per year?” Answers were open ended); health condition (1 item, “Do you have any health conditions that limit the kind of physical activities you could do?” Answers were yes or no; 84.3% answered “no”), physical condition (1 item, “How physically competent are you in sports and outdoor games?” Answers were on a 4-point Likert scale, ranging from “not at all competent” to “very competent”; the average score was 2.7) and alcohol consumption (1 item, “How many drinks of alcohol do you have per day?” Answer were open ended; the average number was 0.84, ranging from 0 to 15). Demographics were also assessed in the survey, including age, gender, race, education, marital status, and employment. These items are reported in Table 2.

On average, the highest addiction quartile individuals had markedly higher use intensity than any other group. However, some of these individuals exhibited low usage and low hedonic well-being from game play. Though less representative of the highest addiction quartile group, the presence of these individuals represents a potential paradox. Their presence in the sample might be attributable to access issues or the application of self-control, whereby individuals might accept a temporary loss of hedonic well-being in an attempt to break behavioral addictive symptoms. Indeed, at any point in time, a fraction of addicted individuals are motivated to quit [30]. Further, withdrawal symptoms from self-regulation would be consistent with the observations of low use and low hedonic well-being in high behavioral addiction individuals.

Conversely, the lowest addiction quartile individuals, while exhibiting the lowest average use profile, did have some individuals who were high-intensity video game players. This also represents an apparent paradox. However, behavioral addiction involves dependence. That some low addiction quartile individuals reported high-intensity use does not necessarily indicate dependence. For example, it may be that these individuals enjoy gaming and have ample opportunity to game (eg, due to a dearth of life responsibilities) but that they could self-regulate usage if life responsibilities dictated the need to do so. While correlated with each other, intense and problematic use of addictive activities are distinct processes [31].

**Table 1:** Summary table of mean survey results by behavioral addiction quartile.

Quartile	Addictive symptoms score^a^, mean (SD)	Time spent on video games per week (hours)	Activities neglected due to video games^b^						Money spent on video games per year (US$)		Hedonic experience in video games (mean score)^c^
			School or work	Sleeping	Eating	Social activities	Physical activities				
1	1.38 (0.22)	13.96	1.25	1.82	1.27	1.25	1.63	200.52		4.36	
2	2.04 (0.19)	17.32	1.72	2.36	1.76	1.74	2.20	236.95		4.27	
3	2.74 (0.20)	21.10	2.24	2.84	2.36	2.49	2.74	290.83		4.29	
4	3.69 (0.48)	28.13	3.05	3.54	3.27	3.39	3.55	817.74		4.39	

^a^Five-point Likert scale (Internet Gaming Disorder Scale).

^b^Five-point Likert scale.

^c^Six-point Likert scale.

**Table 2:** Survey respondent demographics (N=835).

Characteristics			Value
**Gender, n (%)**			
	Male	525 (62.8)	
	Female	310 (37.2)	
Age, average years			32.4
**Race, n (%)**			
	White	608 (72.8)	
	Black	111 (13.3)	
	American Indian/Alaska native	8 (1)	
	Asian or Pacific islander	70 (8.4)	
	Other	37 (4.4)	
	Not applicable	1 (0.1)	
**Education, n (%)**			
	Less than high school	4 (0.5)	
	Highschool	97 (11.6)	
	Some college	249 (29.8)	
	College	352 (42.2)	
	Some graduate school	45 (5.4)	
	Graduate school	88 (10.5)	
**Employment, n (%)**			
	Working	635 (76.1)	
	Looking for a job	70 (8.4)	
	Retired	17 (2)	
	Housewife	34 (4.1)	
	Student	57 (6.8)	
	Other	22 (2.6)	
**Marital status, n (%)**			
	Married	338 (40.5)	
	Single	341 (51.6)	
	Widowed	4 (0.5)	
	Divorced	35 (4.2)	
	Separated	12 (1.4)	
	Married, spouse absent	1 (0.1)	
	Not applicable	14 (1.7)	

## Results

The key variables from the survey are summarized in Table 1. We divided the subjects into 4 balanced groups by self-reported video game addictive symptom (quartiles 1 to 4, with quartile 1 exhibiting the lowest addictive symptoms according to the IGDS). We observed that quartile 4, the group self-reporting the highest addictive symptoms, was the most distinct from its neighboring groups in terms of mean IGDS score, video game time expenditure, video game money expenditure, video game hedonic experience, and neglect of school or work, sleeping, eating, social activities, and physical activities. In other words, quartile-4 players were less bounded to the video game play patterns and costs exhibited by their neighbors in the sorted data. At the individual video game player level, we regressed hedonic experience in video games on the average hours of video game play per week, average hours of video game play per week squared, and a set of variables that served to control for any individual heterogeneity among survey respondents when estimating the relationship of interest. We conducted this regression separately for each addictive symptom quartile (quartiles 1 to 4, in ascending order of addictive symptoms). Video game play per week squared was included to establish the possibility of a nonlinear, quadratic relationship between hedonic experience and video game play intensity (by addictive symptom quartile). Controls included race, education level, primary video game genre, marital status, alcohol consumption, whether the individual played a physical sport, physical condition, and health condition. All interpretations from the model are based on inferential statistical significance testing at standard significance (*α*) levels, as reported in Table 3. The main (nonparenthetical) values in the table represent regression coefficients or marginal effects. They refer to the change in the dependent variable with respect to a unit change in the explanatory variable. The values in parentheses are 2-sided *t* statistics for the regression coefficients.

**Figure 1:**
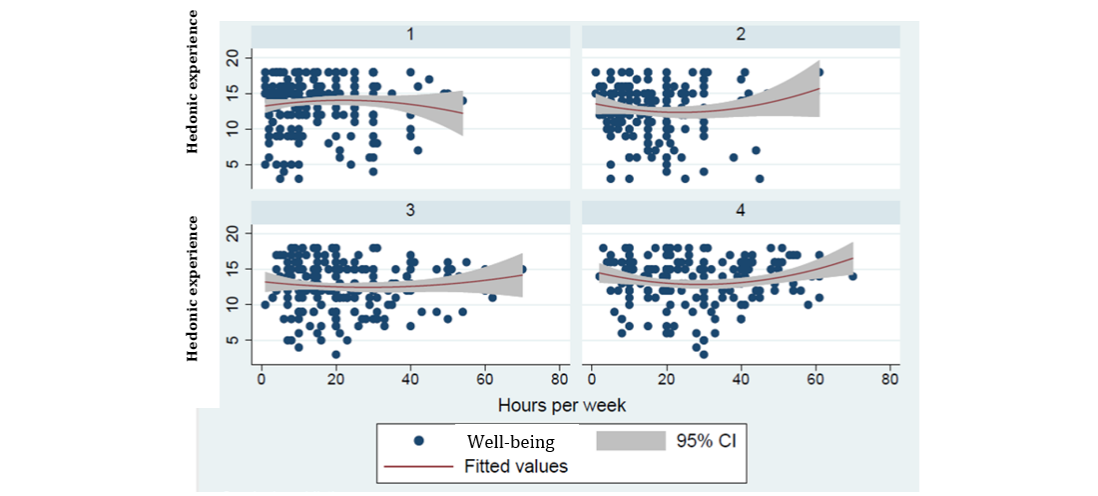
Regression-estimated relationship between hedonic experience and use intensity. Hedonic experience was measured using a 6-point, 3-item Likert scale; thus, total possible hedonic experience was 18 points.

The video game players exhibited a very different hedonic experience in weekly hours played across different addiction quartiles. As addictive quartile rose from quartile 1 to quartile 4, the hours played variables moved from an overall insignificant (not significantly different from flat) relationship with hedonic experience in the quartile 1 and quartile 2 regression results to the predicted U-shaped relationship for quartile 3 and quartile 4. In terms of coefficient value, hours played became smaller for quartiles 1 to 4 and more significant for quartiles 2 to 4 (for quartiles 1, 2, 3, and 4, respectively, *P*=.15, *P*=.17, *P*=.06, and *P*=.01; *t*_216_=1.46, *t*_146_=–1.365, *t*_176_=–1.872, and *t*_155_=–2.589) and hours played squared became larger and more significant (for quartiles 1, 2, 3, and 4, respectively, *P*=.31, *P*=.11, *P*=.03, and *P*=.008; *t*_216_=–1.020, *t*_146_=1.603, *t*_176_=2.131, and *t*_155_=2.676) as we moved from quartile 1 to quartile 4. We found that the higher video game addictive symptom level groups experience a U-shaped (ie, curvilinear) relationship between hedonic experience and intensity of play, whereas groups with lower video game addictive symptom levels exhibited no such relationship. The coefficients for the highest addictive symptom level group (ie, quartile 4) representing marginal effects for hours played per week and hours played per week squared, respectively, were significantly negative (*P*=.005) and significantly positive (*P*=.004). Figure 1 shows scatter plots; the trend curves illustrate the shift toward a U shape from quartile 1 to quartile 4.

The first (low) addiction quartile sample exhibited a trend curve with an inverted U shape. That is, sampled individuals in quartile 1 experienced first increasing, then decreasing hedonic experience with hours played. This trend curve shows that there were diminishing returns to video game play, a common result in microeconomic theory given standard utility functions. These sampled users reported increasing hedonic experience as they become involved in the challenges of a game but at a decreasing rate, until their hedonic experience reached a maximum and descended. The experience of diminishing returns tends to be associated with behavior moderation, as individuals experience negative reinforcement (declining hedonic experience) beyond a certain number of hours played. This observed negative reinforcement may be both a cause and symptom of low reported addiction levels. Moving to quartile 2, quartile 3, and quartile 4, the trend curve between hedonic experience and video game hours played per week became increasingly U shaped (in terms of both coefficient magnitude and coefficient significance level). For quartile 3 and quartile 4, this U-shaped relationship was significant, as reported previously.

From the regression coefficient output related to hours played per week, we can compute the “tipping point” at which, according to prior findings discussed in the introduction, the typical video game player exhibiting addictive symptoms attains the target dopamine response and subsequently faces increasing hedonic experience given additional video play. For quartile 3 players, the estimated minimum hedonic experience value was 26.25 hours per week. For quartile 4 players, the estimated minimum hedonic experience value was 37 hours per week. These postregression estimates suggest that quartile 4 players experienced a longer period of declining hedonic experience, consistent with tolerance theory (ie, “the process whereby increasing amounts of the particular activity are required to achieve the former effects” [12]). The increasing emergence of a U-shaped relationship for higher addiction quartile players suggests that higher-addiction video game players possess a different motivation and experience in video game play. They do not exhibit diminishing returns in play. Rather, they experience decreasing, then increasing hedonic experience. While seeking excessive dopamine release from video games, as found in the prior research, the hedonic experience for these users drops initially as the user plays more. The previous literature suggests that once the user achieves excessive dopamine release, however, the hedonic experience of quartile 3 and quartile 4 users increases. Such significant increases are observed in the regression results we observed. While low addiction quartile players play the game for its own merits, and thus experience a standard diminishing-returns response, the regression results are consistent with the finding that high addiction quartile users play the game to achieve excessive dopamine release and experience an initial “low” period of frustration followed by a “high” period.

In general, there are two elements of addiction that represent theoretical constructs that are significantly consistent with the observed hedonic experience profiles: sensitization and tolerance. Sensitization indicates a hypersensitive reaction to video game exposure [19] and a heightened threshold of video game exposure to experience pleasure [12]. Tolerance makes it more difficult to experience hedonism and pleasure and achieve excessive dopamine release, driving higher addiction quartile users to more average hours of video game use. Sensitization (or amplification) of hedonic experience makes it more difficult for higher addiction quartile individuals to regulate video game use, even during periods in which it may be difficult or represent a high opportunity cost to play until achieving excessive dopamine release [32].

**Table 3:** Regression results, Internet Gaming Disorder Scale addiction score versus use intensity and subject controls.

Variable			Quartile 1, r (*t*)		Quartile 2, r (*t*)		Quartile 3, r (*t*)		Quartile 4, r (*t*)
Hours of video game play, linear regression (hours per week)			0.087 (0.146)		–0.096 (0.174)		–0.105 (0.062)		–0.148 (0.011)
Hours of video game play, quadratic regression (hours per week)			–0.001 (0.309)		0.002 (0.111)		0.002 (0.034)		0.002 (0.008)
**Primary video game genre**									
	Multiplayer Online Battle Arena (0 or 1)	Reference		Reference		Reference		Reference	
	Sports (0 or 1)	–0.989 (0.217)		2.169 (0.034)		0.169 (0.805)		–0.617 (0.280)	
	First person shooting (0 or 1)	0.082 (0.906)		2.260 (0.009)		1.379 (0.031)		–0.954 (0.178)	
	Real time strategy (0 or 1)	0.006 (0.994)		–0.659 (0.753)		–0.917 (0.451)		–1.997 (0.113)	
	Action/adventure (0 or 1)	0.124 (0.855)		2.178 (0.013)		0.718 (0.253)		–1.697 (0.017)	
	Other (0 or 1)	0.829 (0.280)		2.202 (0.027)		2.558 (0.004)		–0.398 (0.745)	
Plays sports (0 or 1)			0.255 (0.611)		–0.133 (0.833)		–0.292 (0.539)		–0.873 (0.576)
Employed (0 or 1)			0.001 (0.995)		0.034 (0.860)		–0.255 (0.105)		–0.068 (0.691)
**Race**									
	White (0 or 1)	0 (0.999)		0 (0.999)		0 (0.999)		0 (0.999)	
	Black (0 or 1)	–1.434 (0.060)		–1.821 (0.029)		–0.497 (0.446)		0.211 (0.700)	
	American Indian or Alaska Native (0 or 1)	0.429 (0.890)		2.126 (0.206)		1.666 (0.550)		3.795 (0.191)	
	Asian or Pacific Islander (0 or 1)	–0.420 (0.651)		–2.571 (0.002)		0.825 (0.257)		–2.220 (0.001)	
	Other (0 or 1)	–2.198 (0.017)		–0.682 (0.626)		–0.364 (0.697)		–1.141 (0.292)	
	Not applicable (0 or 1)	N/A^a^		2.553 (0.437)		N/A		N/A	
**Education**									
	Less than high school (0 or 1)	0 (0.999)		N/A		0 (0.999)		0 (0.999)	
	High school (0 or 1)	0.500 (0.823)		0 (0.999)		–2.980 (0.345)		6.142 (0.053)	
	Some college (0 or 1)	0.116 (0.958)		1.247 (0.150)		–2.722 (0.344)		5.916 (0.056)	
	College (0 or 1)	0.424 (0.846)		2.022 (0.015)		–3.179 (0.313)		6.075 (0.049)	
	Some graduate school (0 or 1)	–0.138 (0.954)		0.057 (0.978)		–3.919 (0.240)		6.606 (0.036)	
	Graduate school (0 or 1)	1.087 (0.627)		3.119 (2.750)		–2.702 (0.397)		6.453 (0.040)	
**Marital status**									
	Married (0 or 1)	0 (0.999)		0 (0.999)		0 (0.999)		0 (0.999)	
	Single (0 or 1)	–.390 (0.001)		–0.380 (0.488)		–1.515 (0.001)		0.012 (0.979)	
	Widowed (0 or 1)	N/A		–2.834 (0.272)		1.422 (0.633)		0.563 (0.850)	
	Divorced (0 or 1)	–2.249 (0.016)		2.604 (0.112)		–3.380 (0.001)		–0.249 (0.855)	
	Separated (0 or 1)	–0.314 (0.886)		–1.279 (0.451)		0.251 (0.889)		–3.512 (0.101)	
	Married, spouse absent (0 or 1)	N/A		N/A		N/A		0.631 (0.834)	
	Not applicable (0 or 1)	–2.741 (0.213)		0.001 (0.999)		1.508 (0.294)		–0.131 (0.933)	
Health condition (0 or 1, with 1 being no limiting health condition)			1.049 (0.027)		0.244 (0.755)		–0.794 (0.046)		–0.650 (0.051)
Physical condition (4-point scale)			1.449 (0.001)		1.064 (0.002)		0.907 (0.001)		0.461 (0.076)
Alcohol consumption per day (drinks per day)			0.231 (0.102)		–0.401 (0.032)		–0.191 (0.200)		–0.078 (0.168)
Constant			7.117 (0.013)		7.616(0.010)		17.050 (0.001)		11.429 (0.001)
lnsigma2 constant			2.172 (0.001)		2.303 (0.001)		2.122 (0.001)		2.050 (0.001)
Observations			249		179		209		188

^a^N/A: not applicable.

## Discussion

The results confirm our two main hypotheses. Namely, for video game players reporting higher behavioral addiction symptoms, time spent on video game play has a significant U-shaped relationship with hedonic experience. For those reporting lower behavioral addiction symptoms, time spent on video game play does not have a significant U-shaped relationship with hedonic experience, but rather a comparatively flat relationship. Hedonic experience is a key motive and consequence for video gaming [8] that can bring subjective happiness and pleasure when playing video games [9]. However, excessive video game play can increase the amount of video game play required to satisfy hedonic needs (ie, tolerance) [10] and lead to development of a hypersensitive reaction to video games (ie, sensitization) [17], affecting hedonic experience in video games and dependency on video games. We confirmed a U-shaped relationship between time spent on video games and hedonic experience among those reporting a higher tendency toward addictive symptoms; we did not see such a relationship for those with a lower tendency. Within the present context, this study is the first to empirically test this U-shaped relationship. We also computed the “tipping point” at which the typical video game user exhibiting addictive symptoms transitions from decreasing to increasing hedonic experience given additional video game play, consistent with what the literature has identified as achievement of target dopamine response. These postregression estimates suggest that higher addiction quartile players experience a longer period of declining hedonic experience, consistent with tolerance theory [10].

The first and second quartile respondents exhibited no substantial addictive symptoms. They are at low risk of developing video game addiction. They exhibited a more typical diminishing-returns response. They are social video gamers or experimental video gamers with limited video game addictive symptoms (excessive time spent, loss of money, and inability to pay attention to their daily responsibilities). Although they may exhibit some video game addictive symptoms, they do not meet the criteria for video game addictive disorder. However, King, Herd, and Delfabbro [33] state that “there is a consistent finding in these types of studies that normal and problem gamers both endorse many of the same motivations for gaming, with problem users simply tending to score much higher than casual users. Thus, the boundary between normal and maladaptive gaming motivations is not always clearly demarcated.”

Nonetheless, results in the third and fourth quartiles show video gamers who display more addictive symptoms (ie, high amount of time spent, high level of tolerance, withdrawal, craving for the behavior, and negative impacts on family, social, and occupational responsibilities). They are frequent players, problematic players, or at-risk players. Their hedonic experience level drops at first (they show disappointment, frustration, and possibly display depressive symptoms), then rises (increasing returns to play). With high tolerance to video game exposure, they are frustrated due to their inability to attain the experience requirements of the game. This behavior is consistent with a study conducted by Kaptsis et al [34], in which they found that problem players exhibited withdrawal symptoms when they did not experience a certain number of requirements of the game. The experiencing of withdrawal symptoms explains the low position of the curve at low usage points for third and fourth quartile players. This indicates negative reactions when deprived of video game exposure. These discomforting withdrawal symptoms include irritability, depressive symptoms, and anxiety. The discomfort of withdrawal symptoms notably induces craving symptoms, such as the need to spend more time playing and the fear of missing specific gaming experiences.

The display of craving symptoms is consistent with a study conducted by Przybylski et al [35]. In this study, the researchers highlighted some psychological components of craving, such as fear of missing social play, novel gaming experiences, and gaming for escape or relaxation as motivating factors for the extensive use of time in gaming. These factors help to explain the rise in hedonic experience among problematic gamers in the third and fourth quartiles. According to King et al [33], this view of craving may explain why problem video game players engage in prolonged, intense, repetitive, or tedious gaming activities. This is consistent with results from our study showing that players with a higher tendency toward addictive symptoms also show higher levels of neglect of other daily activities, such as working or studying, eating, sleeping, and socializing. It is also consistent with multiple previous studies that have reported that problematic video gamers play video games for a prolonged period, skip school or work, experience problems with sleep, and have lower grades at school [36-40]. These symptoms have been observed across different age groups of video game players and other populations [41,42].

### Conclusion

In this study, we conducted a survey of 835 individuals who regularly play video games to determine the relationship between intensity of video game play and hedonic experience of the player. We divided the sample into quartiles by self-reported video game addictive symptom level (using the IGDS) and conducted polynomial regressions separately for each quartile. We found that the higher video game addictive symptom level groups experienced a U-shaped (ie, curvilinear) relationship between hedonic experience and intensity of play, whereas groups with lower video game addictive symptom levels exhibited no such relationship. These results are consistent with sensitization and tolerance theories, which suggest that high-symptom groups are expected to experience frustration and disappointment until achieving excessive dopamine release, at which point their hedonic experience is expected to improve with additional play. Conversely, low-symptom groups experience no such fall-and-rise pattern. This result is consistent with the outcome that members of the latter group play the game for the direct experience, such that their hedonic experience is more directly related to events occurring in the game than to the increasingly elusive pursuit of excessive dopamine release. We also find that high-symptom groups spend substantially more time and money to support video gaming and are much more likely to engage in video gaming at the expense of other important activities, such as working, sleeping, and eating.

### Limitations and Future Research

Although this study shows a novel relationship across all genres of video game play, it does not study the relationship categorically by genre of play. Future research based on longitudinal data can provide information on players and their well-being at different points in time, shedding light on microlevel changes in this relationship. Further, this study does not address cognitive or behavioral consequences of video game play [43]. Lastly, the study relies upon voluntary, self-reported data, which is subject to limitations related to honesty, introspective ability, and sampling (or self-selection) bias. Future studies might rely on random sampling to overcome some of these biases.

## Abbreviations

IGDS: Internet Gaming Disorder Scale
ESA: Entertainment Software Association
